# Ultrasensitive Point‐of‐Care Test for Tumor Marker in Human Saliva Based on Luminescence‐Amplification Strategy of Lanthanide Nanoprobes

**DOI:** 10.1002/advs.202002657

**Published:** 2021-01-06

**Authors:** Shanyong Zhou, Datao Tu, Yan Liu, Wenwu You, Yunqin Zhang, Wei Zheng, Xueyuan Chen

**Affiliations:** ^1^ CAS Key Laboratory of Design and Assembly of Functional Nanostructures, and Fujian Key Laboratory of Nanomaterials Fujian Institute of Research on the Structure of Matter Chinese Academy of Sciences Fuzhou Fujian 350002 China; ^2^ Fujian Science and Technology Innovation Laboratory for Optoelectronic Information of China Fuzhou Fujian 350108 China

**Keywords:** lanthanide nanoprobe, luminescent bioassay, point‐of‐care test, saliva, tumor marker

## Abstract

The point‐of‐care detection of tumor markers in saliva with high sensitivity and specificity remains a daunting challenge in biomedical research and clinical applications. Herein, a facile and ultrasensitive detection of tumor marker in saliva based on luminescence‐amplification strategy of lanthanide nanoprobes is proposed. Eu_2_O_3_ nanocrystals are employed as bioprobes, which can be easily dissolved in acidic enhancer solution and transform into a large number of highly luminescent Eu^3+^ micelles. Meanwhile, disposable syringe filter equipped with nitrocellulose membrane is used as bioassay platform, which facilitates the accomplishment of detection process within 10 min. The rational integration of dissolution enhanced luminescent bioassay strategy and miniaturized detection device enables the unique lab‐in‐syringe assay of tumor marker like carcinoembryonic antigen (CEA, an important tumor marker in clinic diagnosis and prognosis of cancer) with a detection limit down to 1.47 pg mL^−1^ (7.35 × 10^−15^ m). Upon illumination with a portable UV flashlight, the photoluminescence intensity change above 0.1 ng mL^−1^ (0.5 × 10^−12^ m) of CEA can be visually detected by naked eyes, which allows one to qualitatively evaluate the CEA level. Moreover, we confirm the reliability of using the amplified luminescence of Eu_2_O_3_ nanoprobes for direct quantitation of CEA in patient saliva samples, thus validates the practicality of the proposed strategy for both clinical diagnosis and home self‐monitoring of tumor marker in human saliva.

## Graphical abstract

The point‐of‐care detection of tumor markers in saliva is realized based on luminescence‐amplification strategy of lanthanide nanoprobes with disposable syringe filter as bioassay platform. The proposed strategy can be carried out within 10 min, exhibiting a detection limit down to 1.47 pg mL^−1^ for carcinoembryonic antigen, which shows the great promise of salivary tumor marker assay for home self‐monitoring of cancer progression.

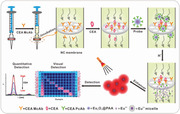

## 1 Introduction

Cancer is one of the major causes of death worldwide.^[^ ^1^ ^]^ In the past decades, tremendous efforts were devoted to the diagnosis and therapy of this disease. Accurate and sensitive assay of tumor markers at the early stage is crucial to decrease the mortality rate.^[^ ^2^ ^]^ Currently, human serum is the most popular and intensively studied diagnostic fluid for the detection of tumor markers.^[^ ^3^ ^]^ As an alternative, saliva, which is an easily accessible fluid, shows prominent potentials for its bioapplication in early cancer diagnosis. Saliva contains several biomarkers including protein, nucleic acid, electrolytes, and hormones, providing important information regarding both oral and systemic health conditions.^[^ ^4^ ^]^ The remarkable advantage of salivary assay lies in the safe and noninvasive collection of saliva, which reduces the potential of cross contamination among healthcare workers and other patients.^[^ ^5^ ^]^ Therefore, saliva is a near‐perfect diagnostic fluid amenable to clinical point‐of‐care (POC) applications.

Saliva contains ≈99.5% water, in which tumor markers are reported to express at concentration much lower than those in human serum.^[^ ^6^ ^]^ Therefore, it remains challenging to realize sensitive and selective salivary analysis. Hitherto, several strategies like enzyme‐linked immunosorbent assay, radioimmunoassay, microarrays, or chromatography have been proposed for the assay of tumor markers in saliva.^[^ ^7^ ^]^ However, due to the extremely low abundance of tumor markers in saliva, the sensitivity or reliability of these techniques is not satisfactory. To address these concerns, it is quite desirable to develop ultrasensitive detection strategy for reliable and rapid assay of tumor markers in saliva. Luminescent bioassay has been proposed as an effective strategy in tumor diagnosis because of its good compatibility with the clinic analytical platforms.^[^ ^8^ ^]^ Specifically, fluorescence intensity change can be used to qualitatively or quantitatively determine the level of tumor markers in saliva as well as other fluids. Nevertheless, the low photoluminescence (PL) intensity of bioprobes and the interference of autofluorescence may severely deteriorate the detection sensitivity and hamper the practicality in salivary assay. To this regard, more and more emphasis has been exerted on developing highly efficient luminescent nanoprobes to achieve better sensitivity. Lanthanide (Ln^3+^)‐activated probes, exhibiting many unique advantages including high physicochemical stability, low toxicity, narrow emission bands, and long PL lifetime, have attracted considerable interest.^[^ ^9^ ^]^ To circumvent the low absorption coefficient of Ln^3+^ ions, organic ligands with large absorption cross sections are utilized to coordinate with Ln^3+^ ions, which can enhance Ln^3+^ luminescence via the antenna effect.^[^ ^10^ ^]^ Such Ln^3+^ chelates are widely employed in dissociation‐enhanced lanthanide fluoroimmunoassay (DELFIA) of different analytes.^[^ ^11^ ^]^ As a potentially superior alternative to conventional Ln^3+^ chelates, Ln^3+^‐doped inorganic nanocrystals (NCs) containing thousands of Ln^3+^ ions can be explored as probes in an effort to significantly increase the labeling ratio of Ln^3+^ per analyte. After introducing the acidic enhancer solution, the initial weakly luminescent Ln^3+^‐doped inorganic NCs are dissolved to release Ln^3+^ ions followed by coordination with sensitization ligands, resulting in a large number of highly luminescent Ln^3+^ micelles. Therefore, the limit of detection (LOD) can be markedly improved. Such dissolution‐enhanced signal amplification strategy is thus extremely promising for assay of trace amount of tumor markers in saliva.

Nowadays, most of the luminescent bioassays are carried out via homogeneous or heterogeneous mode under laboratory conditions where expensive instrumentation and trained personnel are required.^[^ ^12^ ^]^ Homogeneous bioassay is usually a liquid‐phase assay which can be performed through simple ‘‘mix‐and‐read’’ procedures. But the detection sensitivity is critically limited by the energy transfer efficiency between the donors and acceptors.^[^ ^13^ ^]^ By contrast, heterogeneous bioassay exhibits the advantages of high specific recognition and excellent LOD. Nevertheless, tedious separation and washing procedures are usually involved in heterogeneous bioassay. To overcome the limitations of conventional luminescent bioassays, the development of facile assay formats with portable and easy‐to‐operate devices is of great value to enable the patients themselves to monitor tumor markers at an early stage.

**Scheme 1:**
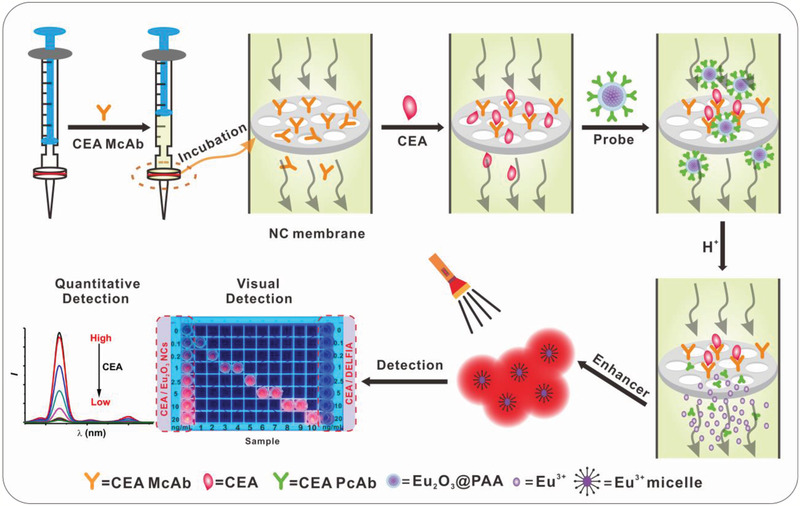
Schematic illustration of CEA detection in saliva. Eu_2_O_3_ NCs and disposable syringe filter equipped with nitrocellulose membrane are employed as nanoprobes and bioassay platform, respectively. The whole assay can be carried out within 10 min, including incubation, labeling, and washing procedures, which allows both quantitatively time‐resolved (TR) and qualitatively visual detection of CEA.

Herein, we report a rapid, noninvasive, and ultrasensitive POC test of tumor marker like carcinoembryonic antigen (CEA) in saliva based on luminescence amplification strategy of lanthanide nanoprobes with disposable syringe filter as bioassay platform (**Scheme** **1**). Benefiting from the excellent dissolution capability of Eu_2_O_3_ in the enhancer solution, an extremely high Eu^3+^ labeling ratio to CEA is achieved, which thus result in an LOD down to 1.47 pg mL^−1^ (7.35 × 10^−15^ m) for CEA. PL intensity change can be visually detected by naked eyes to qualitatively evaluate the CEA level. Significantly, the whole detection process is easy to operate within 10 min, which is much shorter than that of traditionally clinical methods. Furthermore, we demonstrate the feasibility and reliability for both qualitatively and quantitatively determining the level of CEA in patient saliva samples, thus revealing the great promise of the proposed strategy in early POC diagnosis of cancer.

## 2 Results and Discussion

**Figure 1:**
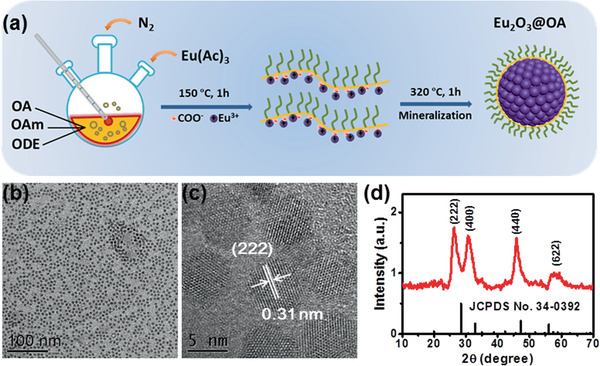
a) Schematic illustration for the synthesis of Eu_2_O_3_ NCs. b) TEM and c) HRTEM images of the as‐prepared Eu_2_O_3_ NCs. d) XRD pattern of the as‐prepared Eu_2_O_3_ NCs. The vertical lines represent the standard pattern of cubic‐phase Eu_2_O_3_ (JCPDS No. 34‐0392).

Eu_2_O_3_ NCs were synthesized from europium acetate precursors via thermal decomposition route in the solvents of oleic acid (OA), oleylamine (OAm), and 1‐octadecene (ODE, **Figure** **1**a).^[^ ^14^ ^]^ To improve the yield of Eu_2_O_3_ NCs, sodium pyrophosphate was used as mineralizer. The as‐prepared spherical Eu_2_O_3_ NCs were highly uniform and monodisperse with average diameter of 5.4 ± 0.5 nm (Figure 1b). The corresponding high‐resolution transmission electron microscopy (HRTEM) image displays a clearly observed lattice spacing of 0.31 nm, which is well coincident with the (222) plane of cubic‐phase Eu_2_O_3_ (Figure 1c). The structure of the as‐prepared sample was also characterized by powder X‐ray diffraction (XRD) analysis (Figure 1d). All the diffraction peaks can be indexed to cubic‐phase Eu_2_O_3_ (JCPDS No. 34‐0392) without other phases, demonstrating the high purity of the as‐synthesized Eu_2_O_3_ NCs. Energy‐dispersive X‐ray spectroscopy (EDX) analysis indicated the existence of Eu and O in the obtained NCs (Figure S1, Supporting Information).

**Figure 2:**
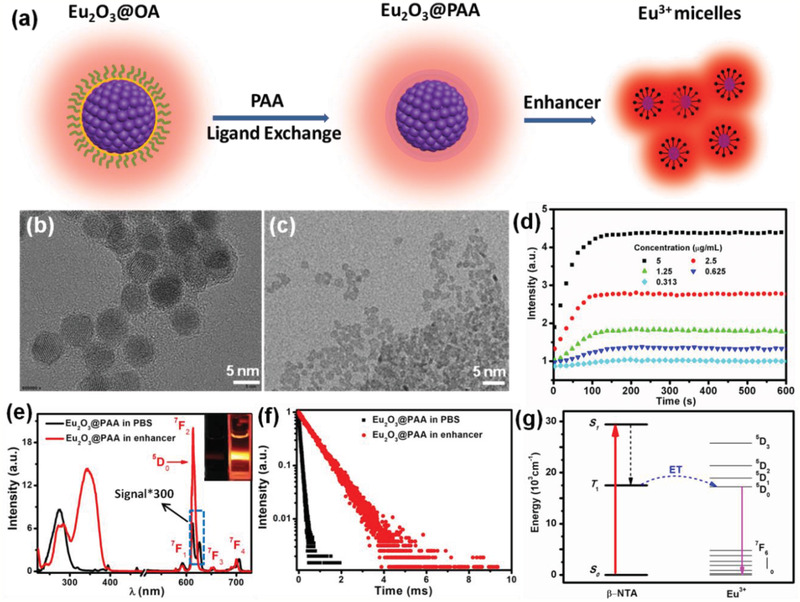
a) Schematic illustration for the synthesis of PAA‐capped Eu_2_O_3_ NCs and Eu^3+^micelles. TEM images of PAA‐capped Eu_2_O_3_ NCs dispersed in b) aqueous solution and c) the enhancer solution. d) Time‐dependent PL signal enhancement behavior of Eu_2_O_3_ NCs with different concentrations in enhancer solution. e) Excitation (left) and emission (right) spectra of PAA‐capped Eu_2_O_3_ NCs dispersed in phosphate buffered saline (PBS) solution and the enhancer solution, respectively. Insets show the PL photographs of PAA‐capped Eu_2_O_3_ NCs dispersed in PBS solution (left) and the enhancer solution (right) upon excitation at 365 nm. f) PL decays of PAA‐capped Eu_2_O_3_ NCs dispersed in PBS solution and the enhancer solution, respectively. g) Schematic energy level diagram showing the *β*‐NTA sensitized PL of Eu^3+^. ET means energy transfer, S_1_ and T_1_ indicate the first excited singlet state and first excited triplet state of *β*‐NTA, respectively.

To render the hydrophobic Eu_2_O_3_ NCs hydrophilic, we modified the surface of OA‐capped NCs with polyacrylic acid (PAA) via ligand exchange (**Figure** **2**a).^[^ ^15^ ^]^ The obtained PAA‐capped Eu_2_O_3_ NCs were highly monodisperse in water (Figure 2b). The zeta potential of the PAA‐capped Eu_2_O_3_ NCs was measured to be −39.11 mV (Figure S2, Supporting Information). The successful capping of PAA on the surface of NCs was further verified by the appearance of carboxyl bands in the Fourier transform infrared (FTIR) spectrum (Figure S3, Supporting Information). To investigate the dissolution behavior of Eu_2_O_3_ NCs, enhancer solution (pH 2.3) containing *β*‐NTA, Triton X‐100, and tri‐*n*‐octylphosphine oxide (TOPO) was added to the aqueous solution of Eu_2_O_3_ NCs, where *β*‐NTA exhibits high affinity and sensitizing ability with Eu^3+^.^[^ ^16^ ^]^ The Eu_2_O_3_@PAA NCs went through a rapid dissolution process in the acidic enhancer solution (Figure 2c). Correspondingly, the PL signal experienced a gradual increase upon addition of the enhancer solution within 3 min (Figure 2d). With increasing the concentration of Eu_2_O_3_ NCs, the final solution exhibited stronger PL signal.

Figure 2e displays the characteristic emission spectrum of PAA‐capped Eu_2_O_3_ NCs in aqueous solution. The emission peaks can be ascribed to the ^5^D_0_→^7^F*_J_* (*J* = 1, 2, 3, and 4) transitions of Eu^3+^ upon excitation at 275 nm. In the corresponding excitation spectrum by monitoring the emission at 613 nm, a broad O^2−^—Eu^3+^ charge transfer absorption from 230 to 310 nm was observed, which is typical for Eu^3+^‐doped oxide hosts. The PL lifetime of ^5^D_0_ was measured to be 0.08 ms (Figure 2f). After addition of the enhancer solution, the resulting solution displayed a broad excitation band peaking at 340 nm and intense sharp emissions originating from the ^5^D_0_→^7^F_0–4_ transitions of Eu^3+^ (Figure 2e). The PL lifetime of ^5^D_0_ for Eu^3+^ micelles (*β*‐NTA–Eu^3+^–TOPO complexes) was measured to be 0.73 ms (Figure 2f), which allows for temporal discrimination from short‐lived (≈ns) background fluorescence.^[^ ^17^ ^]^ Additionally, the absolute luminescence quantum yield of Eu^3+^ micelles was measured to be as high as 45.8%, indicating that (NTA)_3_•(TOPO)_2_ can act as effective light‐harvesting antenna and transfer energy to numerous Eu^3+^ ions released from Eu_2_O_3_ NCs (Figure 2g).^[^ ^18^ ^]^ As such, the dissolved Eu_2_O_3_ NCs in the enhancer solution emitted bright red light under irradiation with a 365‐nm UV lamp, in sharp contrast to the weak emission from Eu_2_O_3_ NCs dispersed in aqueous solution (inset of Figure 2e). Specifically, the PL intensity increased by approximately three orders of magnitude after the Eu_2_O_3_@PAA NCs dissolved in the enhancer solution.

By utilizing the superior PL emission, we explored these Eu_2_O_3_ NCs for the detection of CEA, which is one of the most widely used broad‐spectrum tumor markers.^[^ ^19^ ^]^ Since Martin reported the presence of trace amounts CEA in human saliva,^[^ ^20^ ^]^ more and more efforts unraveled that the level of CEA in human saliva may reflect the state of human health, and the sudden rise of CEA signify the presence of tumor.^[^ ^21^ ^]^ More importantly, it was revealed that the concentration of saliva CEA in patients with oral‐maxillofacial cancer was higher than that in healthy people, demonstrating the significance of saliva CEA for identification of malignant cancer.^[^ ^22^ ^]^ The PAA‐capped Eu_2_O_3_ NCs can be easily connected to CEA polyclonal antibodies (PcAb) by amidation reaction via NHS/EDC coupling method.^[^ ^23^ ^]^ The zeta potential of PcAb‐conjugated Eu_2_O_3_ NCs was found to be −28.29 mV (Figure S2, Supporting Information). The antibody labeling concentration was determined by Coomassie Brilliant Blue method using Bradford kit,^[^ ^24^ ^]^ which indicated that the mass ratio of PcAb/Eu_2_O_3_ was 48 µg mg^−1^ (Figure S4, Supporting Information).

**Figure 3:**
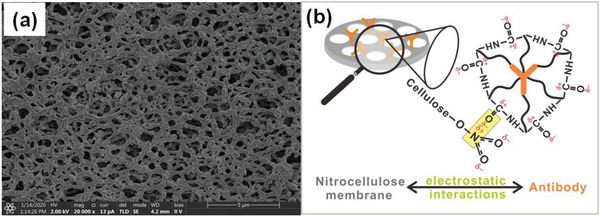
a) Scanning electron microscopy (SEM) image of nitrocellulose membrane, which indicates the homogeneous porous structure with high surface area. b) Schematic illustration for the interaction mechanism between the carbonyl groups of antibody and the nitrate groups of nitrocellulose membrane.

The proposed luminescence‐amplification method allows for a remarkably enhanced sensitivity and noticeably background‐free signal even for relatively low concentrations of analyte. In our work, disposable syringe filter equipped with nitrocellulose membrane was employed as bioassay platform. Nitrocellulose membrane has the homogeneous porous structure with high surface area (**Figure** **3**a), which is suitable for loading and enriching of biomolecules. Note that CEA monoclonal antibody can bind tightly on the nitrocellulose membrane, owing to the strong electrostatic interaction between the nitrocellulose and the hydrophobic parts of antibody (Figure 3b).^[^ ^25^ ^]^ To avoid nonspecific adsorption of other biomolecules, nitrocellulose membrane was then blocked with 2% bovine serum albumin (BSA) in PBS solution (pH 7.4).

**Figure 4:**
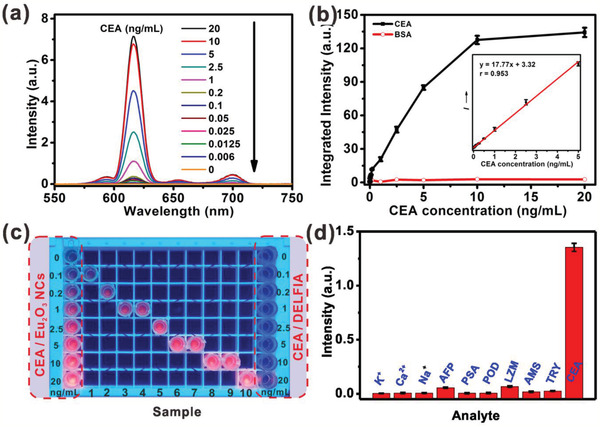
a) TR emission spectra of Eu_2_O_3_ NCs in the enhancer solution with gradient CEA concentrations. b) Calibration curve for the CEA and BSA assay based on integrated TR emission intensity of Eu_2_O_3_ NCs. Inset: the linear range of the integrated TR emission intensity versus the CEA concentration (0.006–5 ng mL^−1^). c) Fluorescence photographs of sample with different CEA concentration upon 365 nm illumination. Fluorescence photographs indicating the standard color cards of CEA standard concentration detected by Eu_2_O_3_ NCs and commercial Eu^3+^‐DTTA DELFIA kit are displayed at the left and right side, respectively. d) Selectivity investigation for the detection of CEA against other interfering analytes. The concentration of each substance was as follows: K^+^ and Ca^2+^: 10 × 10^−3^ m; Na^+^: 160 × 10^−3^ m; alpha‐fetoprotein (AFP), prostate cancer antigen (PSA), peroxidase (POD), lysozyme (LZM), amylase (AMS), and trypsin (TRY): 1 × 10^−6^ m; CEA: 1 × 10^−12^ m. Error bars represent the standard deviations of three independent experiments.

The homogeneous porous structure of nitrocellulose membrane also facilitates the passing through of the unreacted biomolecules and dissolved NCs. As such, all the assay processes can be carried out within 10 min, including incubation, labeling, and washing procedures (Movie S1, Supporting Information), which is ideal for high‐throughput assay of tumor markers in clinical analyses or POC tests. To quantitatively determine the level of CEA, the PL signal was measured on a spectrometer under the TR detection mode (**Figure** **4**a). Upon excitation at 365 nm, red emission from Eu^3+^ micelles was found to be in a gradient increase with increasing the CEA concentration. The integrated PL intensity increased linearly with CEA concentration from 0.006 to 5 ng mL^−1^ (Figure 4b). The LOD,^[^ ^26^ ^]^ defined as the concentration that corresponds to three times the standard deviation above the signal measured in the control experiment by replacing CEA with BSA under otherwise identical conditions (Figure S5, Supporting Information), was determined to be 1.47 pg mL^−1^ (7.35 × 10^−15^ m, Figure 4b). Such an LOD represents more than one order of magnitude improvement than that of the CEA assay based on Ln^3+^ chelates.^[^ ^27^ ^]^ Moreover, upon illumination with a portable 365‐nm UV flashlight, the PL intensity change above 0.1 ng mL^−1^ (0.5 × 10^−12^ m) of CEA can be visually identified according to the fluorescence photographs of standard color card, which enables qualitative evaluating CEA concentrations by naked eyes (Figure 4c). By contrast, based on commercial Eu^3+^‐DTTA DELFIA kit, the PL intensity change even up to 20 ng mL^−1^ of CEA cannot be visually distinguished.

To evaluate the specificity of the assay, we examined the influence of other possible interfering analytes. The control experiment was performed by replacing CEA with carbohydrates, proteins, metal ions, and other tumor markers under otherwise identical conditions. It was observed that the emission signals in control groups were negligibly weak, while for CEA, it resulted in intense fluorescence emission (Figure 4d). Such an exclusively intense fluorescence signal is ascribed to the antibody–antigen binding specificity in the experimental group, confirming the high specificity of the proposed system for CEA detection.^[^ ^28^ ^]^

**Figure 5:**
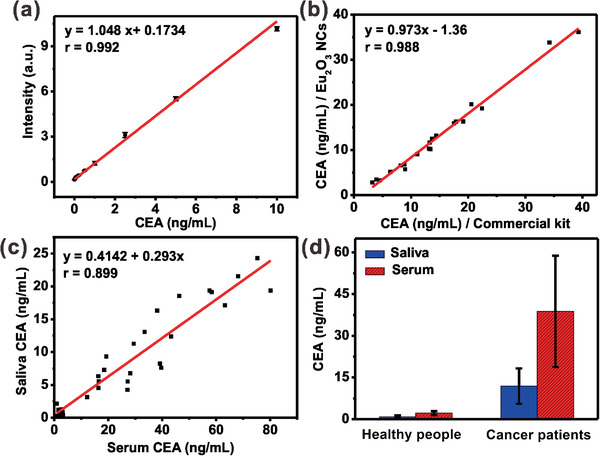
a) Calibration curve for the CEA assay in synthetic saliva based on Eu_2_O_3_ NCs through TRPL assay. b) Correlation of CEA values measured using Eu_2_O_3_ NCs and commercial Eu^3+^‐DTTA DELFIA kit for 20 human saliva samples. c) Correlation of CEA values between saliva and serum samples from 33 volunteers, measured by Eu_2_O_3_ NCs based bioassay system. d) Mean CEA levels (ng mL^−1^) in serum and saliva samples of healthy people and oral cancer patients, respectively. All the saliva and serum samples were diluted ten times with 0.1 m PBS (pH 7.4) for the assay. Three independent experiments were carried out to yield the average value and deviation. The experiments involving human subjects were approved by the Animal Ethics Committee of Fujian Medical University.

It should be noted that high specificity of the proposed system favors the assay of CEA in complex biological fluids like saliva. The calibration curve for the salivary CEA concentration, which was obtained based on synthetic saliva through TRPL assay, displays an excellent linear dependence from 0.05 to 10 ng mL^−1^ (**Figure** **5**a and Figure S6, Supporting Information). Based on this calibration curve, we determined the concentration of CEA in 20 human saliva samples (Table S1, Supporting Information). The determined CEA concentrations were compared with those measured based on commercial Eu^3+^‐DTTA DELFIA kit. As indicated in Figure 5b and Table S1 (Supporting Information), the CEA levels derived from Eu_2_O_3_ nanoprobes are in good accordance with those measured by commercial kit. The correlation coefficient between these two methods was calculated to be as high as 0.988, indicating that the proposed assay system is as reliable as that of commercial kit.

Furthermore, we compared the CEA concentrations in serum and saliva from 22 cases of oral cancer patients and 11 cases of healthy people (Table S2, Supporting Information). As displayed in Figure 5c, the linear correlation coefficient of CEA level between saliva and serum samples was determined to be 0.899, suggesting that CEA levels in saliva are well correlated with those measured in serum from the identical person. The results demonstrated that the CEA levels of saliva samples are much lower than that of serum samples from both cancer patients and healthy people (Figure 5d). The mean value of saliva CEA in 11 healthy individuals was found to be 0.89 ± 0.51 ng mL^−1^, which is lower than that of serum samples (2.19 ± 0.72 ng mL^−1^). Our results also verified that the CEA levels were markedly higher for patient samples than that of the healthy individuals. Specifically, the CEA levels were determined to be 11.87 ± 6.36 and 38.81 ± 20.02 ng mL^−1^ in saliva and serum samples of cancer patients, respectively (Table S2, Supporting Information).

Finally, we investigated the analytical accuracy and precision of our method by determining coefficient of variation (CV) and recoveries. Increasing amounts of CEA were added to two human saliva samples with different initial CEA concentrations. It was found that the CVs of all assays are lower than 7% and the recoveries are in the range of 92–106% (Table S3, Supporting Information). Both parameters well meet the acceptance criteria (CVs ≤ 15%; recoveries in the range of 90–110%) set for bioanalytical method validation.^[^ ^29^ ^]^ These results demonstrate unambiguously the feasibility and reliability for monitoring the level of tumor markers in saliva.

## 3 Conclusion

In conclusion, we have developed a unique lab‐in‐syringe strategy for the assay of tumor marker like CEA in human saliva using amplified luminescence of Ln^3+^‐nanoprobes. This strategy brings together the advantages of the dissolution‐enhanced luminescence amplification for significantly improving the detection limit, and the facile assay procedures with disposable syringe filter for circumventing the cumbersome steps of traditional bioassay approaches. Particularly, by dissolving Eu_2_O_3_ NCs in the *β*‐NTA‐containing enhancer solution, three orders of magnitude amplification of the PL signal from the dissolved NCs was realized. As a result, an excellent detection ability of CEA in saliva with an LOD of 1.47 pg mL^−1^ (7.35 × 10^−15^ m) was achieved. The CEA levels detected in human saliva samples agreed well with those measured by commercial Eu^3+^‐DTTA DELFIA kit, indicating the assay's reliability with a correlation coefficient of 0.988. More importantly, by virtue of the excellent luminescence‐amplification strategy, the PL intensity change can be visually identified above 0.1 ng mL^−1^ (0.5 × 10^−12^ m) of CEA by naked eyes to qualitatively evaluate the CEA levels in saliva. The whole detection process is easy to operate within 10 min, which is highly beneficial for cancer diagnostics by the ordinary people at home. Thus, we anticipate that such a general strategy can be applied for in vitro POC bioassay of a variety of disease markers, which may accelerate the exploitation of lanthanide nanoprobes in versatile diagnostic and therapeutic applications.

## Conflict of Interest

The authors declare no conflict of interest.

## Supporting information

Supporting Information

Supplemental Movie 1
