# Supplementary material for: Ultrasensitive Point‐of‐Care Test for Tumor Marker in Human Saliva Based on Luminescence‐Amplification Strategy of Lanthanide Nanoprobes
Source: Adv Sci (Weinh). 2021 Jan 6;8(5):2002657. doi: 10.1002/advs.202002657 (PMC7927602; doi:10.1002/advs.202002657)
Supplement: Supplementary file 1 — Supporting Information [file ADVS-8-2002657-s001.pdf]

© 2020 Wiley-VCH GmbH

Supporting Information

for *Adv. Sci.,* DOI: 10.1002/advs.202002657

Ultrasensitive Point-of-Care Test for Tumor Marker in Human Saliva Based on Luminescence-Amplification Strategy of Lanthanide Nanoprobes

*Shanyong Zhou, Datao Tu, Yan Liu, Wenwu You, Yunqin Zhang, Wei Zheng, and Xueyuan Chen**

Ultrasensitive Point-of-Care Test for Tumor Marker in Human Saliva Based on Luminescence-Amplification Strategy of Lanthanide Nanoprobes

*Shanyong Zhou,[‡] Datao Tu,[‡] Yan Liu, Wenwu You, Yunqin Zhang, Wei Zheng, and Xueyuan Chen**

S. Y. Zhou, Dr. D. T. Tu, Dr. Y. Liu, Dr. W. W. You, Y. Q. Zhang, Prof. W. Zheng, Prof. X. Y. Chen
CAS Key Laboratory of Design and Assembly of Functional Nanostructures, State Key Laboratory of Structural Chemistry, and Fujian Key Laboratory of Nanomaterials, Fujian Institute of Research on the Structure of Matter, Chinese Academy of Sciences, Fuzhou, Fujian 350002, China

S. Y. Zhou, Dr. D. T, Tu, Dr. Y. Liu, Prof. W. Zheng, Prof. X. Y. Chen
Fujian Science & Technology Innovation Laboratory for Optoelectronic Information of China, Fuzhou, Fujian 350108, China

[‡] These authors contribute equally.

* E-mail: xchen@fjirsm.ac.cn

Experimental Procedures

Chemicals and materials: $EuAc_3 \cdot 4H_2O$, oleic acid (OA), oleylamine (OM), and 1-octadecene (ODE) were purchased from Sigma-Aldrich (China). $Na_4P_2O_7$, NaCl, $NaHCO_3$, Na_2CO_3, NaOH, KCl, $CaCl_2$, K_2HPO_4, Na_2HPO_4, $KHCO_3$, $MgCl_2$, citric acid, sodium polyacrylate, cyclohexane, diethylene glycol (DEG), ethanol, acetate, β-NTA, Triton X-100, tri-n-octylphosphine oxide (TOPO), hydrochloric acid, Tween20, bovine serum albumin (BSA), polyacrylic acid (PAA), N-hydroxysuccinimide (NHS), and 1-ethyl-3-(3-dimehylaminopropyl) carbodiimide hydrochloride (EDC) were bought from Sinopharm Chemical Reagent Co., China. Carcinoembryonic antigen (CEA), CEA monoclonal antibody (McAb, coating), and CEA polyclonal antibodies (PcAb, labeling) were supplied by Shanghai Linc-Bio Science Co. Dissociation enhanced lanthanide fluoroimmunoassay (DELFIA) kit based on Eu^{3+}-DTTA complex for CEA assay was provided by Daan Gene Co., Ltd. of Sun Yat-Sen University, China. Disposable syringe filter and nitrocellulose membrane (0.45 mm) were purchased from Jiangsu Green Union Science Instrument Co., Ltd., China. The 96-well Nunc Immobilizer Amino plates were bought from Thermo Fisher Scientific Inc. All the chemical reagents were used as bought without further purification. Distilled water was used throughout.

Characterization: Transmission electron microscopic (TEM) images were recorded on a JEOL-2010 TEM equipped with the energy dispersive X-ray (EDX) spectrum. Scanning electron microscope (SEM) measurement was performed by using a JSM6700F SEM. Powder X-ray powder diffraction (XRD) patterns were collected using an X-ray diffractometer (MiniFlex2, Rigaku) with Cu Kα1 radiation (λ = 0.154 nm). The Zeta potentials were measured with a Malvern Instrument (Nano ZS ZEN3600). Fourier-transform infrared (FTIR) spectra were recorded on a Magna 750 FTIR spectrometer. All fluorescence measurements were identified by a spectrometer equipped with both continuous (450 W) xenon and pulsed flash lamps (FLS980, Edinburgh Instruments).

Synthesis of Eu_2O_3 nanocrystals (NCs): Eu_2O_3 NCs were synthesized from europium acetate precursors via a facile thermal decomposition route.[1] Briefly, 1 mmol of $EuAc_3 \cdot 4H_2O$ were added into a 100 mL three-neck round-bottom flask containing 5 mL of OA, 5 mL of oleylamine, and 10 mL of 1-octadecene. The flask was then degassed with N_2 for 10 min at room temperature (RT). After that, the reaction solution was heated to 150 °C for 1 h to dissolve the reactants and remove water. Subsequently, 1 mmol of $Na_4P_2O_7$ were added to the solution. After 10 min, the temperature was increased to 320 °C and maintained for 1 h, resulting in the formation of the Eu_2O_3 NCs. The mixture was cooled down to RT. The Eu_2O_3 NCs were precipitated by addition of ethanol, collected by centrifugation, washed with cyclohexane and ethanol several times, and finally re-dispersed in cyclohexane.

Synthesis of PAA-capped Eu_2O_3 NCs: We modified the surface of OA-capped NCs with polyacrylic acid (PAA) via ligand exchange.[2] 8.0 mL of DEG containing 0.5 g PAA was heated to 110 °C with vigorous stirring under N_2 flow. Then, 15 mL of cyclohexane solution containing 30 mg Eu_2O_3 NCs was injected to the hot solution. The mixture was heated to 240 °C and kept at this temperature for 1 h until the solution became clear. After the solution was cooled down to RT, excess dilute hydrochloric aqueous solution was added. Finally, PAA-capped Eu_2O_3 NCs were obtained by centrifugation, washed with pure water. The resulting powders can be well dispersed in water by ionizing the carboxylic groups with a dilute NaOH solution.

Calculation of photoluminescence (PL) quantum yield (QY): The absolute PLQY was measured at RT by employing a barium sulfate coated integrating sphere (150 mm in diameter, Edinburgh) as the sample chamber that was mounted on the FLS980 spectrometer with the entry and output port of the sphere located in 90° geometry from each other in the plane of the spectrometer (excited by 365 nm light from xenon lamp). The PL emission intensity from 550 to 750 nm was integrated to calculate the absolute PLQY. A standard tungsten lamp was used to correct the optical response of the instrument. All the spectral data were corrected for the spectral response of both the spectrometer and the integrating sphere. We calculated the absolute PLQY based on the following equation:

$$PLQY = \frac{N_e}{N_a} = \frac{L_s}{E_r - E_s}$$

where N_e and N_a are the photons emitted and absorbed, respectively; L_s is the emission intensity; E_r and E_s are the intensities of the excitation light in the presence of the pure hexane solution (reference) and the Eu^{3+} micelles (sample) dispersed in hexane solution, respectively.

Synthesis of CEA PcAb-conjugated Eu_2O_3 NCs: The PAA-capped Eu_2O_3 NCs can be easily connected to CEA PcAb by amidation reaction via NHS/EDC coupling.[3] In a typical process, 5 mg of sodium polyacrylate was dispersed in 1 mL aqueous solution containing 10 mg PAA-capped Eu_2O_3 NCs. The mixture was stirred for 1 h at RT. After washing with distilled water for three times, 1 mL of PBS containing 1 mg NHS and 5 mg EDC was added for the activation of carboxyl with constant stirring for 30 min. Thereafter, 0.5 mg of CEA PcAb was added and incubated for 30 min. The resulting labeled NCs were purified by washing with PBS for three times and stored at 4 °C.

Preparation of the enhancer solution: We prepared the enhancer solution with a modified formula as previously reported.[4] β-NTA and TOPO with final concentrations of 15 μM and 50 μM, respectively, were mixed in 100 mL of aqueous solution (pH 2.3) containing 1 % (m/v) acetate and 0.1 % (m/v) Triton X-100.

Preparation of synthetic saliva: Synthetic saliva was prepared according to the reported method.[5] 10 mL of aqueous solution containing K_2HPO_4 (0.025 M), Na_2HPO_4 (0.024 M), $KHCO_3$ (1.57 M), NaCl (0.1 M), $MgCl_2$ (0.002 M), 6 mL of citric acid (0.025 M), and 10 mL of $CaCl_2$ (0.15 M) were mixed together. The pH was adjusted to 7.0 with NaOH (5 M) or HCl (12 M) solutions and the volume was quantitatively made up to 100 mL. The mixture was sterilized by autoclaving. When the ambient temperature was achieved, 1 mL of aqueous solution containing α-amylase (1 g/L) from human saliva and lysozyme (0.1 g/L) from chicken egg white were added with constant stirring for 30 min.

Preparation of human saliva and serum samples: The human saliva and serum samples from healthy donors or patients with cancer were provided by Union Hospital Affiliated to Fujian Medical University, Fuzhou, China. The extraction of saliva sample was conducted on people without eating, drinking, smoking or chewing gum within 30 min. After chewing the cotton swabs for medical use, the cotton swabs were placed into the centrifuge tube and centrifuged with the speed of 2000×g to obtain the saliva samples. The saliva sample was diluted 10 times with 0.1 M pH 7.4 PBS before further experiments. The human serum samples were obtained from coagulated human blood by centrifugation at 1000×g for 10 min, also diluted 10 times with 0.1 M pH 7.4 PBS before further experiments. All experiments involving human subjects were approved by the Animal Ethics Committee of Fujian Medical University.

Coating syringe filter with CEA McAb: Syringe filter was employed as a solid-phase reactor combined with nitrocellulose membrane which was rinsed by carbonate buffer solution (pH 9.6), and then incubated with 20 μg/mL CEA McAb overnight at 4°C. After washing with PBST (0.05% Tween in PBS solution) for three times, the nitrocellulose membrane was incubated in blocking buffer (2% BSA in PBS solution, pH 7.4) overnight, dried and finally assembled into disposable syringe filter for further use.

Quantitatively time-resolved detection of CEA: CEA standard solution was diluted into different concentrations by PBS buffer. Then, 0.5 mL of CEA standard solution was rapidly sucked and injected into the syringe filter by 1 mL injector, incubated for 1 min and rinsed with 0.5 mL of PBST for three times to remove excess CEA. After that, 0.5 mL of CEA PcAb labeled Eu_2O_3 NCs (2 μg/mL) were rapidly introduced into the syringe filter by 1 mL injector, incubated for 1 min and rinsed again with 0.5 mL of PBST for three times. After washing, 0.5 mL of enhancer solution was rapidly added into the syringe filter. After 3 min, the filtrate was subjected to centrifuge tube. 200 μL of filtrate was taken by injector into 96-well plate, and the PL signal was measured on a multimodal

microplate reader under the time-resolved (TR) detection mode. The excitation wavelength was 365 nm. The delay time was set to be 200 μs. The calibration curve of CEA concentration and fluorescence intensity was established upon various CEA concentrations. The assay of human saliva or serum samples was conducted following the same procedure by simply replacing the CEA standard solution with human saliva or serum samples. Every measurement was repeated three times and the CEA levels in human saliva or serum samples were determined by the calibration curve.

Qualitatively visual detection of CEA: The incubation, labeling, washing and dissolution processes for CEA assay were conducted following the same procedure mentioned above. Upon excitation with a 365-nm UV flashlight, standard color card indicating CEA standard concentration was established with their corresponding fluorescence photographs. The assay of human saliva or serum samples was conducted following the same procedure by simply replacing the CEA standard solution with human saliva or serum samples. The CEA levels in human saliva or serum samples were evaluated according to the standard color card.

Statistical Analysis: All the assay experiments were repeated thrice independently unless otherwise indicated. Statistical analysis was compiled on the means of the data obtained from at least three independent experiments. The data of all assays were presented as the mean value ± standard deviation (SD). “n” numbers for each experiment are indicated in the figure legends.

Table S1. Comparison of the CEA levels in 20 human saliva samples independently determined by the luminescence-amplification strategy based on Eu_2O_3 NCs and the commercial kit based on Eu^{3+}-DTTA complex, respectively. The unit of the CEA levels is ng/mL. The CEA levels derived from Eu_2O_3 nanoprobes are in good accordance with those measured by commercial kit, indicating that the proposed assay system is as reliable as that of commercial kit. Three independent experiments were carried out to yield the average value and deviation.

Samples	Eu_2O_3 NCs	Commercial kit	Samples	Eu_2O_3 NCs	Commercial kit
**1**	3.51±0.31	3.91±0.31	**11**	33.81±2.37	34.21±2.73
**2**	11.65±0.93	13.25±0.79	**12**	15.92±1.27	17.52±1.40
**3**	10.20±0.61	13.4±0.81	**13**	5.76±0.52	8.96±0.81
**4**	9.10±0.728	11.1±0.99	**14**	6.82±0.48	8.82±0.62
**5**	5.16±0.46	6.36±0.31	**15**	3.29±0.29	4.49±0.41
**6**	2.82±0.14	3.22±0.22	**16**	20.15±1.81	20.55±1.85
**7**	12.5±0.87	13.7±1.09	**17**	13.21±1.18	14.41±1.15
**8**	10.32±0.93	13.12±0.91	**18**	16.33±1.31	19.13±1.34
**9**	19.21±1.53	22.41±2.01	**19**	36.12±3.25	39.32±3.53
**10**	16.26±1.31	17.86±1.42	**20**	6.59±0.59	8.19±0.74

Table S2. Results of CEA level in serum and saliva samples from 11 healthy people and 22 oral cancer patients based on Eu_2O_3 NCs. The unit of the CEA levels is ng/mL. It is revealed that the CEA levels of saliva samples are much lower than that of serum samples from both cancer patients and healthy people. Meanwhile, the CEA levels were significantly increased in patients samples relative to that of the healthy individuals. Three independent experiments were carried out to yield the average value and deviation.

Healthy people	Serum	Saliva	Cancer patients	Serum	Saliva	Cancer patients	Serum	Saliva
**1**	2.33±0.15	0.59±0.04	**12**	19.31±1.54	9.34±0.65	**23**	16.48±1.15	5.54±0.44
**2**	1.59±0.12	1.24±0.09	**13**	29.45±2.65	11.29±0.91	**24**	27.14±1.89	4.26±0.25
**3**	3.14±0.28	0.89±0.05	**14**	38.14±2.66	16.32±1.13	**25**	75.24±4.51	24.31±2.18
**4**	2.26±0.18	0.63±0.06	**15**	46.29±3.24	18.56±1.09	**26**	80.16±5.61	19.38±1.55
**5**	0.89±0.07	2.15±0.17	**16**	58.34±4.08	19.15±1.72	**27**	12.18±0.97	3.16±0.22
**6**	1.28±0.11	0.33±0.02	**17**	68.15±4.77	21.55±1.94	**28**	16.35±1.14	4.56±0.36
**7**	2.67±0.24	1.26±0.11	**18**	16.34±0.98	6.34±0.38	**29**	28.17±2.25	6.78±0.61
**8**	3.27±0.26	0.39±0.03	**19**	18.52±1.29	7.29±0.58	**30**	39.63±3.56	7.62±0.53
**9**	2.46±0.19	0.45±0.03	**20**	27.18±1.63	5.53±0.48	**31**	43.28±3.46	12.38±0.99
**10**	1.59±0.13	1.06±0.08	**21**	33.46±2.67	13.08±1.17	**32**	57.54±4.02	19.37±1.74
**11**	2.59±0.18	0.85±0.07	**22**	39.16±3.13	8.26±0.66	**33**	63.27±3.79	17.13±1.19

Table S3. Assay precision and analytical recovery of CEA added to two saliva samples. The coefficient of variations (CVs) of all assays are lower than 7% and the recoveries are in the range of 92-106%. Both parameters well meet the acceptance criteria (CVs ≤ 15%; recoveries in the range of 90-110%) set for bioanalytical method validation.[6] Four independent experiments were carried out to yield the average value and deviation. These results demonstrate unambiguously the feasibility and reliability for monitoring the level of tumor markers in human saliva.

Added (ng/mL)	Found (ng/mL)	CV (%) (n=4)[a]	Recovery (%)
Sample 1	4.61±0.31	6.85	——
0.50	5.1±0.29	5.77	98.0
10.7	14.52±0.51	3.48	92.67
56.0	63.75±2.74	4.31	105.60
Sample 2	20.54±1.22	5.94	——
0.50	21.71±1.08	4.96	103.18
10.7	30.04±1.84	6.14	96.16
56.0	78.25±4.14	5.29	102.23

[a] Intra-assay using four different wells in a plate.

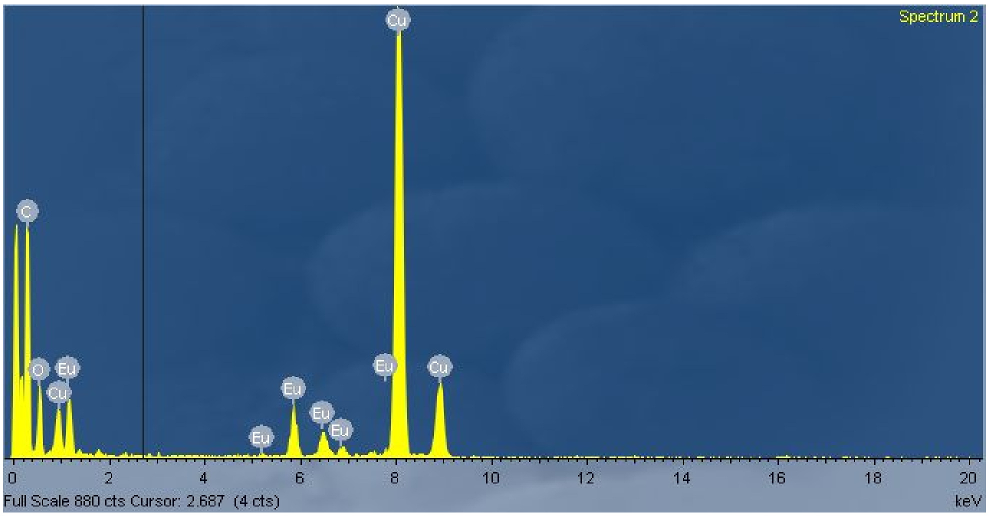

Figure S1. EDX spectrum of the as-prepared Eu_2O_3 NCs, confirming the existence of Eu and O in the obtained NCs.

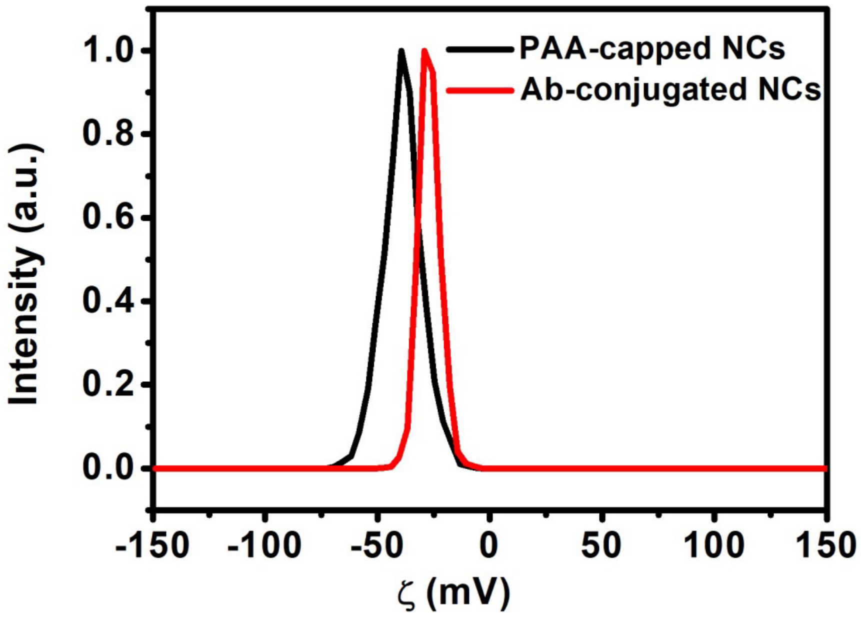

Figure S2. ζ-potentials of the PAA-capped and CEA Ab-conjugated Eu_2O_3 NCs. The zeta potential value of the PAA-capped Eu_2O_3 NCs was measured to be -39.11 mV. For the Ab-conjugated Eu_2O_3 NCs, the zeta potential was determined to be -28.29 mV.

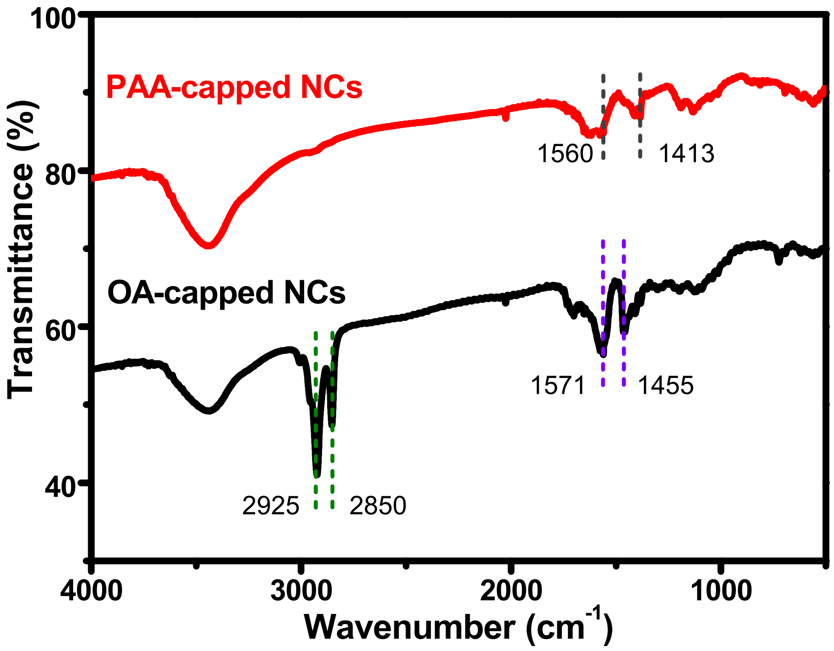

Figure S3. FTIR spectra of OA-capped and PAA-capped Eu_2O_3 NCs, respectively. After ligand exchange, the original stretching vibrations of methylene in the long alkyl chain peaking at 2925 and 2850 cm^{-1}, and the stretching vibrations of carboxyl peaking at 1571 and 1455 cm^{-1} disappeared. These results indicate the successful removal of oleate ligands from the surface of OA-capped NCs. Meanwhile, the stretching vibrations of carboxyl peaking at 1560 and 1413 cm^{-1} appear, which confirm the successful capping of PAA on the surface of NCs.

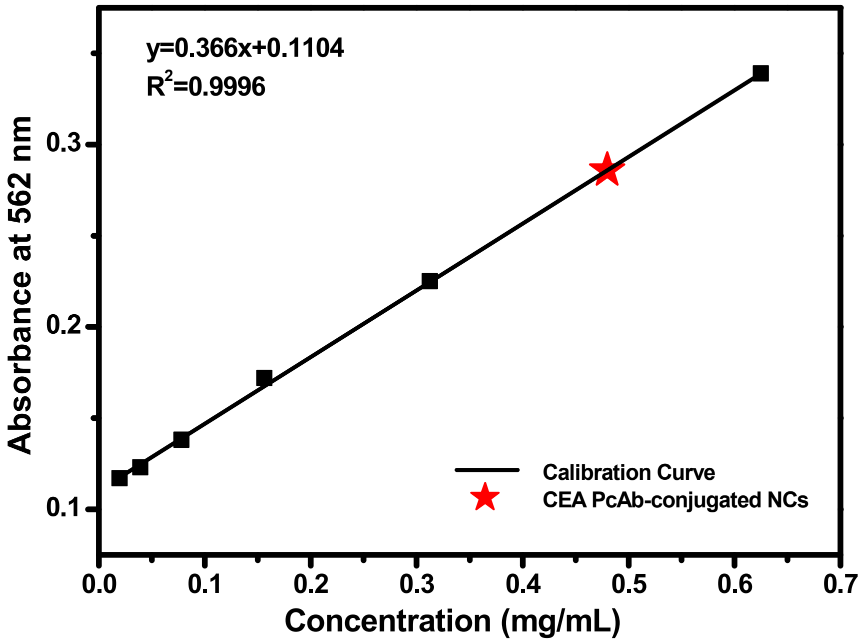

Figure S4. Quantitative analysis of CEA PcAb conjugated to the Eu_2O_3 NCs. The antibody labeling concentration was measured by Coomassie Brilliant Blue method using Bradford kit.[7] 10 mg of CEA PcAb-conjugated Eu_2O_3 NCs were dissolved in 1 mL PBS (pH 7.4, 0.1 M) for the measurement. According to the calibration curve of absorbance at 562 nm corresponding to analysis of different concentrations of antibody, the CEA PcAb concentration labeling on Eu_2O_3 NCs was determined to be 48 μg/mg.

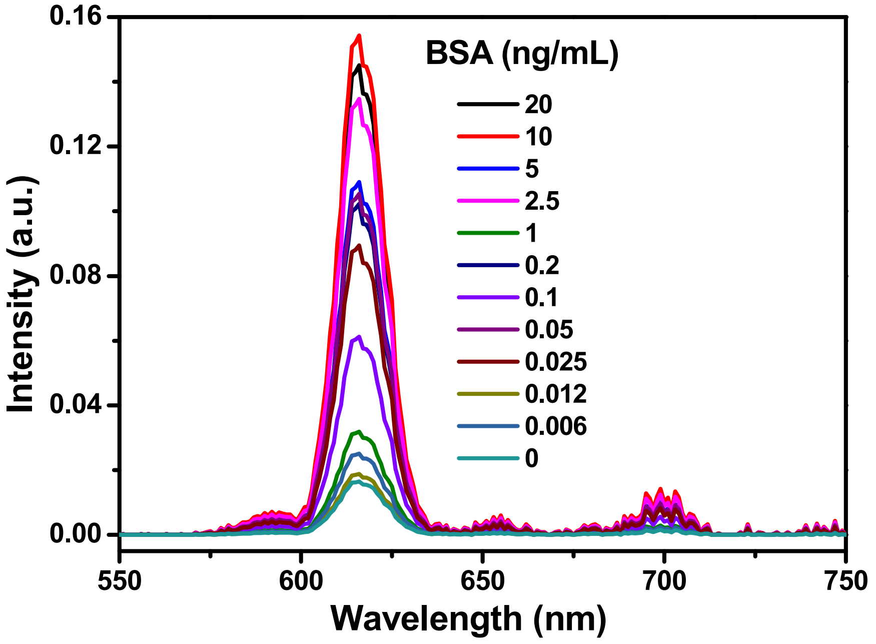

Figure S5. TR emission spectra of Eu_2O_3 NCs in the enhancer solution for the control experiment by replacing CEA with BSA under otherwise identical conditions. Statistical analysis was compiled on the means of the data obtained from three independent experiments. Upon excitation at 365 nm, the PL intensity of the the control experiment was found to be much lower than that of CEA assay.

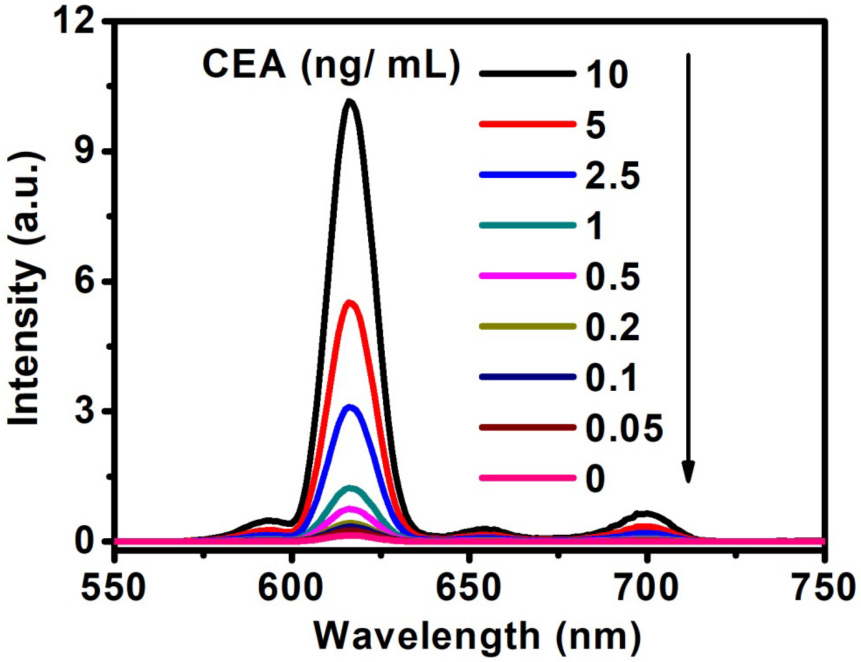

Figure S6. TR emission spectra of Eu_2O_3 NCs in the enhancer solution for the assay of CEA in the synthetic saliva upon excitation at 365 nm. The red emission from Eu^{3+} micelles was found to be in a gradient decrease along with the decreased CEA concentration.

Supporting Video Captions

Movie S1. Detailed procedures of CEA detection based on luminescence-amplification strategy of lanthanide nanoprobes.

References

[1] D. Y. Wang, Y. J. Kang, X. C. Ye, C. B. Murray, *Chem. Mater.* **2014**, *26*, 6328.

[2] T. R. Zhang, J. P. Ge, Y. X. Hu, Y. D. Yin, *Nano Lett.* **2007**, *7*, 3203.

[3] W. W. You, D. T. Tu, W. Zheng, X. Y. Shang, X. R. Song, S. Y. Zhou, Y. Liu, R. F. Li, X. Y. Chen, *Nanoscale* **2018**, *10*, 11477.

[4] I. Hemmila, S. Dakubu, V. M. Mukkala, H. Siitari, T. Lovgren, *Anal. Biochem.* **1984**, *137*, 335.

[5] S. Viswanathan, C. Rani, A. V. Anand, J. A. A. Ho, *Biosens. Bioelectron.* **2009**, *24*, 1984.

[6] J. N. Hao, Y. S. Li, *Adv. Funct. Mater.* **2019**, *29*, 1903058.

[7] H. H. Guo, X. R. Song, W. Lei, C. He, W. W. You, Q. Z. Lin, S. Y. Zhou, X. Y. Chen, Z. Chen, *Angew. Chem. Int. Ed.* **2019**, *58*, 12195.
